# **No Association of nineteen***COX-2*** gene variants to preclinical markers of atherosclerosis The Cardiovascular Risk in Young Finns Study**

**DOI:** 10.1186/1471-2350-13-32

**Published:** 2012-07-02

**Authors:** Kati Lähteelä, Tarja Kunnas, Leo-Pekka Lyytikäinen, Nina Mononen, Leena Taittonen, Tomi Laitinen, Johannes Kettunen, Markus Juonala, Nina Hutri-Kähönen, Mika Kähönen, Jorma S Viikari, Olli T Raitakari, Terho Lehtimäki, Seppo T Nikkari

**Affiliations:** 1 Department of Medical Biochemistry, University of Tampere Medical School, Tampere, Finland; 2 Department of Clinical Chemistry, University of Tampere and Tampere University Hospital, Tampere, Finland; 3 Department of Pediatrics, University of Oulu, Oulu Finland and Department of Pediatrics, Vaasa Central Hospital, Vaasa, Finland; 4 Department of Clinical Physiology, University of Kuopio, and University of Eastern Finland, Kuopio, Finland; 5 FIMM, Institute for Molecular Medicine Finland, Helsinki, Finland; 6 Department of Medicine, University of Turku and Turku University Hospital, Turku, Finland; 7 Department of Pediatrics, University of Tampere and Tampere University Hospital, Tampere, Finland; 8 Department of Clinical Physiology, University of Tampere and Tampere University Hospital, Tampere, Finland; 9 Research Centre of Applied and Preventive Cardiovascular Medicine, University of Turku and Department of Clinical Physiology, University of Turku and Turku University Hospital, Turku, Finland

## Abstract

**Backgroud:**

The role of cyclooxygenase-2 (*COX-2*) single nucleotide polymorphisms has mostly been studied in relation to advanced atherosclerosis, but little is known how they contribute to preclinical disease. In the present study we analyzed whether *COX-2* gene variants associate independently with the early subclinical markers of atherosclerosis, carotid intima-media thickness and carotid artery distensibility in a population of young healthy Caucasian adults.

**Methods:**

SNPs for association analysis were collected from the *COX-2* gene and 5 kb up- and downstream of it. There were 19 SNPs available for analysis, four genotyped and fifteen imputed. Genotype data was available for 2442 individuals participating in the Cardiovascular Risk in Young Finns Study. Genotype imputation was performed using MACH 1.0 and HapMap II CEU (release 22) samples as reference. Association analysis was performed using linear regression with an additive model. PLINK was used for true genotyped SNPs and ProbABEL for imputed genotype dosages. False discovery rate was used to take into account multiple testing bias.

**Results:**

Two of the *COX-2* variants (*rs689470, rs689462*) associated with distensibility (p = 0.005) under the linear regression additive model. After adjustment with gender, age, body mass index and smoking status, association between these SNPs and distensibility remained significant (p = 0.031). Subjects carrying the minor alleles had higher value of carotid artery distensibility compared to the major allele homozygotes. However, after correcting p-values for multiple testing bias using false discovery rate, association was lost. Another *COX-2* variant *rs4648261* associated with mean carotid intima-media thickness (p = 0.046) and maximal carotid intima-media thickness (p = 0.048) in the linear regression model. Subjects carrying the minor allele of *rs4648261* had lower values of mean and maximal carotid intima-media thickness compared to subjects homozygote for major allele. After adjustments the associations were lost with both mean and maximal carotid intima-media thickness. Thus, no statistically significant associations of the studied *COX-2* variants with carotid artery distensibility or carotid intima-media thickness were found.

**Conclusions:**

Our results suggest that in a Finnish population, there are no significant associations between *COX-2* variants and early atherosclerotic changes in young adulthood.

## Background

Atherosclerosis is the main pathologic process underlying cardiovascular disease. The chronic inflammatory reaction in vessel wall has an important role in disease initiation and progression [1].

Inflammation is partly regulated by prostaglandins which are produced through the enzyme cyclooxygenase (COX). Cyclooxygenase catalyzes the rate-limiting steps in prostaglandin production from arachidonic acid. *COX-1* is constitutively expressed in most human tissues under basal conditions. *COX-2* expression is primarily induced in response to inflammatory stimuli by growth factors, mitogens, and cytokines [2].

The important role of *COX-1* expression in platelets leading to atherothrombosis has been clarified through its inhibition with acetosalicylic acid. However, the role of *COX-2* activity in these events is still unclear [3]. *COX-2* expression has been detected in endothelial cells, smooth muscle cells, monocytes, and macrophages within human atherosclerotic lesions [4,5]. Prostaglandins produced by the COX-2 route may have impact on the development of atherosclerosis by influencing inflammatory reaction, platelet function, leukocyte-endothelial cell adhesion and smooth muscle cell proliferation and migration [6].

The role of *COX-2* has previously been studied in relation to advanced atherosclerosis with clinical complications, such as coronary artery disease [7,8] myocardial infarction and ischemic stroke [9]. However, atherosclerosis begins in childhood and progresses for decades until clinical complications such as myocardial infarction appear [10].

Contributions of genetic alterations would be expected to manifest themselves in younger rather than in older subjects. In fact, it has been suggested that low COX-2 level may enhance foam cell production and consequently, increase the risk of atherosclerosis and cardiovascular disease [11]. Functional studies have been made for only two of the tested SNPs (rs 20417, rs 5275), but this does not necessarily mean that the others are not functional [12]‐[14]. There are 302 known SNPs in the COX-2 gene available from the NCBI dbSNP human database. In the present study we analyzed all the 19 *COX-2* SNPs available from the HapMap II CEU (release 22) and their association with subclinical markers of carotid atherosclerosis, such as carotid intima-media thickness (CIMT) and carotid artery distensibility (Cdist).

## Methods

### Subjects

The Cardiovascular Risk in Young Finns Study is a five-center longitudinal cohort study of atherosclerosis risk factors from childhood to adulthood [15], which has been described in detail previously [16,17]. The first cross-sectional survey involving 3596 subjects between the ages of 3 and 18 years was conducted in 1980. The latest follow-up was done in 2007. Genotype data for the present analysis was available for 2442 individuals. All subjects gave their written informed consents in 2001 and the local ethics committees in all five centers (Helsinki, Kuopio, Oulu, Tampere and Turku) approved the study.

### Clinical characteristics and risk factors

Height and weight were measured, smoking habits and history of diabetes were assessed with a questionnaire. A random zero sphygmomanometer (Hawksley &Sons Ltd, Lancing, UK) was used for blood pressure measurements. The mean of three measurements was used in the analysis. For the determination of serum lipids and apolipoprotein B and A1 levels, venous blood samples were drawn after an overnight fast. Lipid concentrations and glucose were measured using standard methods [18]. LDL cholesterol concentration was calculated according to Friedewald formula. Fasting plasma highly sensitive C-reactive protein (hs-CRP) concentrations were analyzed by latex turbidometric immunoassay (Wako Chemicals GmbH, Neuss, Germany).

### Ultrasound measurements

Ultrasound examinations for baseline measure were performed using Sequoia 512 ultrasound mainframes between September 2001 and January 2002 (Acuson, Mountain View, CA) with a 13.0 MHz linear array transducer, as previously described [16]. The left carotid artery was scanned according to a standardized protocol. The image in the carotid intima-media thickness measurements was focused on the posterior wall of the left carotid artery. A magnified image was recorded from the angle showing the greatest distance between the lumen-intima interface and the media-adventitia interface. A minimum of four measurements of the common carotid far wall were taken 10 mm proximal to the bifurcation in order to derive mean CIMT. The between-visit (two visits 3 months apart) coefficient of variation for CIMT measurements was 6.4 %.

Several moving-image clips of the beginning of the carotid bifurcation and the common carotid artery with duration of 5 seconds were acquired and stored in digital format for subsequent offline analysis. To assess carotid artery distensibility indices, the best-quality cardiac cycle was selected from the 5-second image clips. The common carotid diameter 10 mm from carotid bifurcation was measured from the B-mode images with ultrasonic calipers at least twice in end diastole and end systole, respectively. The means of the measurements were used as the end-diastolic and end-systolic diameters. Ultrasound and concomitant brachial blood pressure measurements were used to calculate carotid distensibility (Cdist = [(Ds - Dd)/Dd]/(Ps - Pd), where Ds is systolic diameter, Dd is diastolic diameter, Ps is systolic blood pressure, and Pd is diastolic blood pressure). Cdist measures the ability of arteries to expand as a response to pulse pressure caused by cardiac contraction and relaxation. To assess reproducibility of ultrasound measurements, 57 subjects were reexamined 3 months after the initial visit (2.5 % random sample). The between-visit coefficient of variation was 2.7 % for carotid artery diastolic diameter and 16.3 % for Cdist [19].

### Genotyping and imputation

Genomic DNA was extracted from peripheral blood leukocytes using a commercially available kit and Qiagen BioRobot M48 Workstation according to the manufacturer’s instructions (Qiagen, Hilden, Germany). Genotyping was done for 2556 samples using custom build Illumina Human 670 k BeadChip atWelcome Trust Sanger Institute. Genotypes were called using Illuminus clustering algorithm. 56 samples failed Sanger genotyping pipeline QC criteria (i.e., duplicated samples, heterozygosity, low call rate, or Sequenom fingerprint discrepancy). From the remaining 2500 samples one sample failed gender check, three were removed due to low genotyping call rate (< 0.95) and 54 samples for possible relatedness (pi-hat > 0.2). 11766 SNPs were excluded based on Hardy-Weinberg equilibrium test (p ≤ 1e-06), 7746 SNPs failed missingness test (call rate < 0.95) and 34596 SNPs failed frequency test (minor allele frequency < 0.01). After quality control there were 2442 samples and 546677 genotyped SNPs available for further analysis [20].

Genotype imputation was performed using MACH 1.0 [21] and HapMap II CEU (release 22) samples as reference. After imputation there were 2543887 imputed SNPs available. SNPs with squared correlation between imputed and true genotypes ≥ 0.30 were considered well imputed.

### Statistical analysis

Phenotypes mean CIMT, maximal CIMT and Cdist were transformed to normality using Box-Cox transformation. Standard residuals were extracted from the model and outliers ≥ 4 SD were removed. SNPs for association analysis were collected from the *COX-2* gene and 5 kb up- and downstream of it (chromosome 1, pos 186,635,945-186,654,559). There were 19 SNPs available for analysis, four genotyped and fifteen imputed. Association analysis was performed using linear regression with additive and recessive models. PLINK [22] was used for true genotyped SNPs and ProbABEL [23] for imputed genotype dosages. To take into account multiple testing bias introduced by testing 19 SNPs simultaneously, we used false discovery rate (FDR) [24] to correct the p-values. Q values (= corrected p-values) less than 0.05 were considered significant. FDR calculations were done using R language package ‘fdrtool’ [25].

## Results

Background characteristics of the study population are shown in Table 1. The participants´ mean age was 31.4 years at examination in 2001. Nineteen SNPs included in the study are presented in Table 2. Data on *COX-2* variants associated with markers of early phase atherosclerosis is presented in Table 3. Imputation quality was high. For *rs689470*, *rs689462* and *rs4648261* the squared correlation between imputed and true genotypes (Rsq) values were 0.999, 1 and 0.690 respectively.

**Table 1:** Background characteristics of the study subjects.

	Mean	SD	N
Gender (male/female)	1123 (46.0 %)/1319 (54.0 %)		2442
Age (year)	31.4	5.0	2442
BMI (kg/m^2^)	25.1	4.4	2276
CIMT (mm)	0.58	0.09	2081
Cdist (%/10 mmHg)	2.17	0.75	2071
SBP (mmHg)	116.7	13.1	2084
DBP (mmHg)	70.8	10.8	2084
Total cholesterol (mmol/l)	5.1	1.0	2103
HDL cholesterol (mmol/l)	1.3	0.3	2101
LDL cholesterol (mmol/l)	3.3	0.8	2075
Triglycerides (mmol/l)	1.3	0.9	2103
Apolipoprotein B (g/l)	1.1	0.3	2103
Apolipoprotein A1 (g/l)	1.5	0.3	2103
hs-CRP (mg/l)	1.9	4.0	2103
Glucose (mmol/l)	5.1	0.9	2103
Smoking (yes/no)	511 (23.6 %)/1650 (76.4 %)		2161

SBP = systolic blood pressure, DBP = diastolic blood pressure.

**Table 2:** ***COX-2*variants included in the study.**

Variant	Minor/major allele	Minor allele frequency
Genotyped and imputed		
rs2206593	T/C	0.074
rs5275	C/T	0.317
rs10911905	G/T	0.084
rs2143417	T/G	0.100
Imputed		
rs4648304	T/C	0.009
rs689470	T/C	0.016
rs2066826	A/G	0.077
rs5277	C/G	0.174
rs4648262	T/G	0.0001
rs4648261	A/G	0.069
rs2745557	T/C	0.174
rs20417	C/G	0.100
rs689466	G/A	0.205
rs689462	C/A	0.016
rs12042763	A/C	0.287
rs2745559	T/G	0.174
rs11583191	T/C	0.081
rs2143416	G/T	0.111
rs2179555	G/A	0.088

**Table 3:** **Association of the*COX-2*variants with CIMT, Cdist and cholesterol values.**

Phenotype	Variant	Minor/major	P	P	Q	Measured mean value			Number of individuals			
		allele	unadjusted	adjusted†	adjusted ††	hommajor	het	hom minor	hom major	het	hom minor	total
Mean carotid intima-media thickness (mm)	rs4648261	A/G	0.046	0.116	0.896	0.58	0.57	0.53	1583	99	4	1686
Max carotid intima-media thickness (mm)	rs4648261	A/G	0.048	0.121	0.477	0.62	0.61	0.56	1583	99	4	1686
Carotid distensibility (% /10 mmHg)	rs689470	T/C	0.005	0.031	0.137	2.17	2.42		2000	71		2071
	rs689462	C/A	0.005	0.031	0.137	2.17	2.42		2000	71		2071
Total cholesterol (mmol/l)	rs5275	C/T	0.014	0.007	0.017	5.20	5.11	5.05	955	930	208	2093
LDL cholesterol (mmol/l)	rs5275	C/T	0.009	0.006	0.022	3.31	3.24	3.16	940	921	204	2065

† linear regression model, adjusted with gender, age, BMI and smoking status.

†† false discovery rate corrected p-values (= q-values) adjusted with gender, age, BMI and smoking status.

hom major = major allele homozygote, het = heterozygote, hom minor = minor allele homozygote.

*Rs689470* and *rs689462* were in 100 % linkage disequilibrium. The minor *T* allele of *rs689470* was in linkage with the minor *C* allele of *rs689462*. These SNPs associated with carotid artery distensibility (p = 0.005) under the linear regression additive model (Table 3). After adjustment with gender, age, body mass index (BMI) and smoking status, association between these SNPs and Cdist remained significant (p = 0.031). Subjects with the minor *T* allele of the *rs689470* and the minor *C* allele of the *rs689462* had higher value of Cdist compared to the major allele homozygotes. However, after correcting p-values for multiple testing bias, association disappeared (q = 0.137).

Another *COX-2* variant *rs4648261* associated with mean CIMT (p = 0.046) and maximal CIMT (p = 0.048) under the linear regression model. Subjects carrying the minor *A* allele of *rs4648261* had lower values of mean CIMT and maximal CIMT compared to subjects presenting major allele *GG* genotype. After adjustment with gender, age, BMI and smoking status the associations were lost with both mean CIMT (p = 0.116) and maximal CIMT (p = 0.121). Also, after correcting for multiple testing bias, the associations were non-significant with mean CIMT (q = 0.896) as well as with maximal CIMT (q = 0.477).

One of the studied SNPs associated with serum cholesterol values (Table 3). Subjects with the minor *C* allele of the *rs5275* had lower mean values of total cholesterol (p = 0.014) and LDL cholesterol (p = 0.009) compared with the major *T* allele. After adjustment with gender, age, BMI and smoking status, associations remained significant with total cholesterol (p = 0.007) and LDL cholesterol (p = 0.006). Moreover, associations remained significant after multiple testing correction with total cholesterol (q = 0.017) as well as with LDL cholesterol (q = 0.022).

Finally, we also tested a recessive model in evaluating the effect of SNPs on the markers of atherosclerosis. The results were similar in both the additive and recessive model. After adjustment with gender, age, BMI, smoking status, and multiple testing correction in the recessive model, the only associations that remained significant were total cholesterol (q = 0.014) and LDL cholesterol (q = 0.041) for rs 5275.

## Discussion

We evaluated 19 single nucleotide polymorphisms in the *COX-2* gene for association with early structural and functional changes of atherosclerosis. No significant associations were found between SNPs under analysis and early atherosclerotic changes in our study.

Decreased Cdist can be considered as an early sign of functional atherosclerosis. It has been approved as an independent cardiovascular risk factor and predictor for mortality in patients with advanced renal disease [26]. There are no previous studies of associations of *COX-2* SNPs with Cdist and few that report their association with other markers of preclinical atherosclerosis in young subjects.

The minor *C* allele of the *rs20417* has been associated with atherosclerotic plaques [7,27]. In contrast, Orbe et al (2006) found that CIMT was reduced in the *rs20417* minor *C* allele carriers. Moreover, Cipollone et al (2004) reported that the minor *C* allele of the *rs20417* was associated with reduced risk of myocardial infarction (MI) and stroke [9]. Other studies have not been able to replicate the result for MI and stroke [28]. The *rs20417* was not associated with CIMT in our study population.

Concerning subclinical atherosclerosis in coronary, carotid and aortic arterial beds, Rudock et al (2009) investigated eight *COX-2* SNPs and their relationship to calcified plaques in subjects with type 2 diabetes mellitus [27]. They found that three SNPs (*rs689466rs2066826* and *rs20417*) were associated with coronary or carotid calcified plaques. These three variants were included in our study but they did not associate with CIMT parameters or Cdist. Moreover, we could not do a subgroup analysis, since our study included only 24 subjects who had type 1 diabetes. Since CIMT and Cdist are significantly associated with high LDL-cholesterol, elevated blood pressure, obesity and smoking [16,19], it is obvious that genetics, e.g. the variation in COX-2 genotype, may be of only minimal importance and difficult to reveal.

Studies showing association with cardiovascular disease for several other SNPs included in this study have already been published. For the minor *C* allele of *rs5275* a beneficial role has been proposed [8,29]. For *rs689466* minor *G* allele, there is a report of no effect [8] and a beneficial effect [27]. *Rs2066826* minor *A* allele has also had an beneficial effect [27]. None of these SNPs were associated with CIMT or Cdist in our study.

Subjects with the minor *C* allele of *rs5275* had lower mean values of total cholesterol and LDL cholesterol compared with the major *T* allele. The difference was small and not clinically relevant. However, in line with our results, there is a previously reported interaction of *rs5275* and alcohol in relation to plasma cholesterol levels, where the minor allele carriers with low alcohol intake had the lowest lipid levels [8].

A major limitation of the present study is that the minor allele frequency of many *COX-2* variants was very low. For *rs689470* and *rs689462* it was 0.016. Therefore, there were no rare allele homozygotes present in our data and the frequencies of the wild type and heterozygote genotypes were 0.97 (n = 2000) and 0.03 (n = 71) respectively. Minor allele frequency of *rs4648261* was slightly better, 0.069. Still, there were only four homozygotes for the minor allele and the frequencies of the wild type and heterozygote genotypes were 0.94 (n = 1583) and 0.06 (n = 99) respectively. The number of subjects for minor allele homozygotes and heterozygotes is too small to obtain valid results in association study concerning such a complex disease as atherosclerosis.

## Conclusions

In conclusion, our results suggest that in a Finnish population, there are no significant associations between *COX-2* variants and early atherosclerotic changes in young adulthood.

## Competing interests

The authors declare that they have no competing interests.

## Authors' contributions

KL contributed to the analysis and interpretation of the data and drafting the manuscript. TK and STN contributed to conception and design of this study, drafting the manuscript and revising the article critically for important intellectual content. LPL contributed to the analysis and interpretation of the data. OTR, MJ, NH-K, JSV, TLe, MK, LT, TLa and JK contributed to the conception and design of this study and revising the article critically for important intellectual content. NM and JK contributed revising the article critically for important intellectual content. All authors read and approved the final manuscript.

## Pre-publication history

The pre-publication history for this paper can be accessed here:

http://www.biomedcentral.com/1471-2350/13/32/prepub
